# Behavioral risk factors associated with reported tick exposure in a Lyme disease high incidence region in Canada

**DOI:** 10.1186/s12889-022-13222-9

**Published:** 2022-04-22

**Authors:** Cécile Aenishaenslin, Katia Charland, Natasha Bowser, Esther Perez-Trejo, Geneviève Baron, François Milord, Catherine Bouchard

**Affiliations:** 1 Groupe de Recherche en Épidémiologie des Zoonoses et Santé Publique (GREZOSP), Faculté de médecine vétérinaire, Université de Montréal, Saint-Hyacinthe, QC Canada; 2 Centre de recherche en santé publique de l’Université de Montréal et du CIUSSS du Centre-Sud-de-l’Île-de-Montréal, Montréal, QC Canada; 3 École de santé publique de l’Université de Montréal, Montréal, Canada; 4 Direction de la santé publique, CIUSSS de l’Estrie-CHUS, Sherbrooke, Canada; 5 Département des sciences de la santé communautaire, Faculté de médecine et des sciences de la santé, Université de Sherbrooke, Sherbrooke, Canada; 6 CISSS de la Montérégie-Centre, Longueuil, Canada; 7 Public Health Risk Sciences Division, National Microbiology Laboratory, Public Health Agency of Canada, Saint-Hyacinthe, QC Canada

**Keywords:** Tick bites, Tick exposure, Ticks, Tick-borne diseases, Lyme disease, Prevention, Preventive behaviors, Risk factors

## Abstract

**Background:**

Tick-borne diseases, and especially Lyme Disease (LD), are on the rise in Canada and have been met with increasing public health concern. To face these emerging threats, education on the prevention of tick bites remains the mainstay of public health intervention. The objective of this study was to assess the adoption of preventive behaviors toward tick bites and LD and to investigate the association between behavioral risk factors and reported tick exposure in a Canadian, LD high incidence region (Estrie region, Quebec, Canada).

**Methods:**

A cross-sectional study was conducted in 2018 which used a telephone questionnaire administered to a random sample of 10,790 adult residents of the study region. Questions investigated tick exposure, LD awareness, attitudes towards LD risk, outdoor and preventive behaviors, as well as antibiotic post-exposure prophylaxis (PEP) treatments in the case of a tick bite. Descriptive and multivariable analyses were carried out, considering the nine administrative subregions and the stratified survey design.

**Results:**

The sub-regional prevalence of reported tick exposure in the previous year ranged from 3.4 to 21.9%. The proportion of respondents that adopted preventive behaviors varied from 27.0% (tick checks) to 30.1% (tick repellent) and 44.6% (shower after outdoor activities). A minority of respondents (15.9%) that sought healthcare after a tick bite received a PEP treatment. Performing tick checks (Odds ratio = 4.33), time spent outdoors (OR = 3.09) and living in a subregion with a higher public health LD risk level (OR = 2.14) were associated with reported tick exposure in multivariable models.

**Conclusions:**

This study highlights the low level of adoption of preventive behaviors against tick bites in a region where LD risk is amongst the highest in Canada. This suggests a concerning lack of improvement in LD prevention, as low levels of adoption were already reported in studies conducted in the last decade. Innovative and evidence-based approaches to improve education on ticks and tick-borne diseases and to promote behavior changes are urgently needed in Canada.

**Supplementary Information:**

The online version contains supplementary material available at 10.1186/s12889-022-13222-9.

## Background

Climate change and modifications in land use are altering the distribution, survival and behaviors of multiple tick species in North America, which can carry human and animal pathogens [1]. In North America, Lyme Disease (LD) is primarily caused by *Borrelia burgdorferi* sensu stricto and transmitted by the blacklegged tick, *Ixodes scapularis,* in the Eastern regions. It remains the most frequently reported tick-borne disease, with an estimation of 476,000 human infections annually in the United States [2]. The northward expansion of tick populations has also generated a rapid emergence of the disease in Canada. Between 2009 and 2018, the number of reported cases increased by a factor of 10, from 144 to 1487, in this country [3]. Other tick-borne diseases are also on the rise and create new public health concerns in Canada and North America, including anaplasmosis, babesiosis, Powassan encephalitis and *Borrelia miyamotoi* disease [1]. To address these known and emerging threats, the prevention of tick bites remains the mainstay of any public health intervention.

In Canada, LD endemic areas are locations where transmission to humans of *B. burgdorferi* by resident populations of vector ticks has been confirmed by active or passive surveillance [4]. The number of recorded LD endemic areas has risen from one area in Ontario in the 1990s to numerous areas in several other provinces including Quebec, Nova Scotia, New Brunswick, Manitoba and British-Columbia [5, 6]. From 2014 to the present, the extent of known endemic areas is much wider in terms of number and geographic range [4]. The emergence of *I. scapularis* in Canada may be related to climate change, the dispersal of ticks by migratory birds, change in land use (i.e. the reforestation of agricultural areas) and the increase of the white-tailed deer population [7]. In the Quebec province, the first *I. scapularis* established tick populations were detected in 2008 [8, 9] and there are now several established tick populations in southern Quebec [10].

Primary recommended public health measures to prevent tick bites and tick-borne diseases rely on individual behaviors, including wearing protective clothing, the use of tick repellents on clothing and skin, taking a shower or bath after an activity in a risk area, and regular tick checks, ie. the practice of a body examination to quickly detect and remove ticks on or attached to the skin [11]. Some Canadian LD endemic regions now also offer the possibility to receive a post exposure prophylactic (PEP) treatment with one dose of doxycycline after a bite, depending on certain criteria, to prevent infection with LD [12]. At the peri-domestic level, regular mowing of the lawn, collection of dead leaves and other measures aimed at reducing tick habitats near homes are also recommended [13].

Evidence demonstrating the effectiveness of these behaviors to reduce LD risk is still scarce and inconsistent [14–19]. In a systematic review and meta-analysis of factors affecting tick bites and tick-borne diseases, Fischoff et al. (2019) showed that both environmental and behavioral risk factors seem to significantly impact the risk of tick bites in the United States and Canada [20]. This meta-analysis also revealed that each individual preventive behavior was associated with reduced risk for tick bites and tick-borne diseases. However, very few studies have investigated the adoption and effectiveness of these behaviors in Canada, where the risk of tick bites and tick-borne diseases is emerging and varies greatly across the country [11, 21].

The main objective of this study was to assess the adoption of preventive behaviors toward tick bites and LD and to investigate the association between behavioral risk factors and tick exposure in a Canadian LD high incidence region.

## Methods

### Study region

**Fig. 1:**
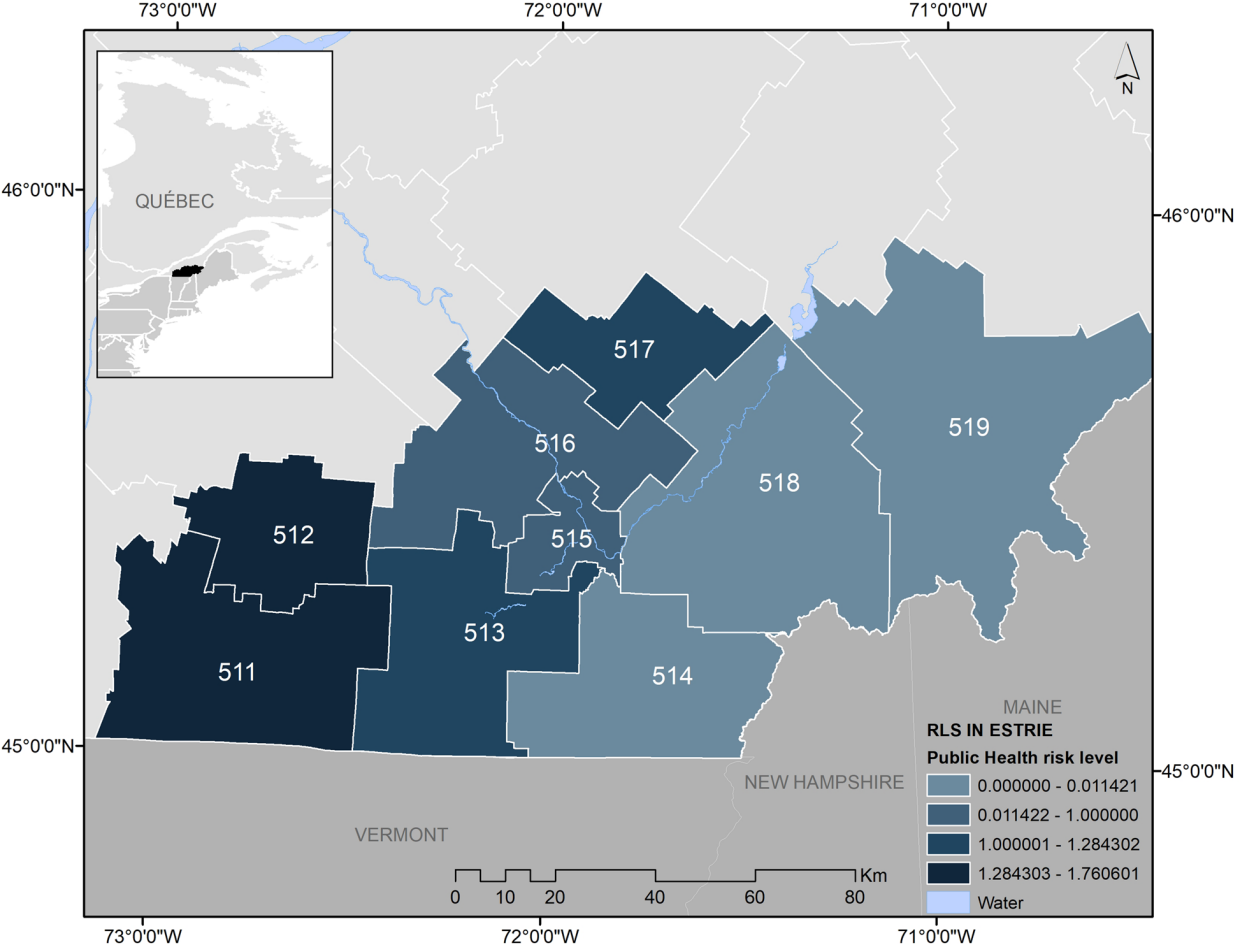
RLS in the Estrie region showing the distribution of Lyme disease public health risk level. RLS names: La Pommeraie (511), Haute-Yamaska (512), Memphrémagog (513), Coaticook (514), Sherbrooke (515), Val Saint-François (516), Asbestos (517), Haut-Saint-François (518), Granit (519)

This cross-sectional study was conducted in the Estrie region, an administrative area located in the southeast of the Quebec province in Canada, which borders the states of Maine and Vermont in the U.S. (Fig. 1). The region totals 10,197 km^2^ and was home to 483,722 people in 2018 [22]. The Estrie region has the highest number of reported LD human cases in Quebec, with an estimated incidence of 41.6 cases per 100,000 inhabitants in 2019, which is more than 4 times higher than the the second most affected region in the province [23]. The Estrie region is divided into nine health subregions called *Réseaux locaux de services* (RLS), which are numbered from 511 to 519 (Fig. 1). Known LD risk is higher in western RLS, as illustrated by the publicly available indicator of municipality-level risk of acquiring LD determined by the Institut national de santé publique du Québec (INSPQ), herein referred to as the *public health risk level* [24]. Values range from 0 (possible risk) to 2 (significant risk). This indicator combines the incidence of LD cases in the past 5 years, the number of ticks submitted to the passive acarological surveillance system and the presence of the three developmental stages of *I. scapularis* (larvae, nymph and adult) and of infected ticks, detected with the active acarological surveillance system [24].

### Data collection

In 2018, the public health department of Estrie (*Direction de santé publique de l’Estrie*) conducted a general populational health survey, which included 19 questions and sub-questions regarding tick bites and LD prevention (questionnaire available in Supplementary material 1). A random sample of adult (> 18 years old) residents of the region was stratified by population density of each health subregion (RLS, Fig. 1). The questionnaire was administered in French or English to residents of the Estrie region by an external survey firm using telephone interviews from June to November 2018. Questions used for this study measured tick exposure over the previous 12 months, LD awareness, level of concerns towards LD risk, outdoor behaviors (time spent outdoors for primary occupation, practice of hiking, gardening, camping), frequency of adoption of preventive behaviors for tick bites and tick-borne diseases (use of tick repellent, showering and tick checks), and PEP treatments following a tick bite. In addition, the survey collected information on socio-demographic factors, including the respondents’ postal code, and whether the residence was in proximity (within 150 m) to forests, woods or tall grass. Sampling weights were created based on age, sex and RLS strata. The respondents’ postal codes were used to determine both the municipality of respondents and the corresponding health subregion (RLS). Since each subregion contains one or more municipalities, a public health risk marker of LD risk for the subregions was computed by averaging the public health risk level for municipalities (2018 status) within the RLS (Fig. 1).

### Statistical analysis

Analyses were restricted to respondents that knew of LD. Data on the frequency of adoption of preventive behaviors (tick repellent, showering and tick checks) were dichotomized for further analysis: respondents reporting having applied a behavior often or always over the last 12 months were considered as having adopted the behavior, and those reporting applying it never or rarely were considered as having not adopted it. The sampling weights were applied to all descriptive analyses, except frequencies and in the initial description of the sample. Choropleth maps at the RLS and municipality level, were prepared for the public health risk level value and for prevalence of reported tick exposure. Chi-squared tests with the adjusted Wald statistic were used to test the relationship between two categorical variables.

Inference on the association between behavioral risk factors and reported tick exposure by the respondents (adjusting for spatial heterogeneity and socio-demographic confounders) was carried out in two ways: with a mixed-effects logistic regression model including random effects for RLS (without applying sampling weights) and with a quasi-binomial model with logit link, accounting for the stratified survey design. Variables of primary interest were included in all multivariable models. These were time spent outdoors for primary occupation, practice of hiking, gardening, camping, adoption of tick repellent, showering and tick checks. All models controlled for public health risk index at the residency location and whether the respondent’s home was near a high-risk area. Additional potential confounders were age, sex, and education. Model selection was based on subject matter expertise and the literature rather than statistical criteria. However, we assessed the importance of confounders by determining whether their inclusion changed the odds ratios of the other variables by more than 10% [25].

All analyses were carried out with R software version 4.1.0 and R library “survey”, version 4.0 [26]. Maps were created with ArcGIS version 10.6.1.

## Results

A total of 10,790 participants was recruited for the study, which corresponds to a response rate of 40%. The sample description, in terms of subregions (RLS), sex, age and education, is presented in Table 1. Of the 10,790 study respondents, 10,410 (96.0%) knew of LD with 75.2% (*n* = 7427) being aware of the risk of acquiring LD in their municipality (13.1% reported not being at risk, 11.6% did not know). Of those aware of LD, 809 (9.6%) reported that they or a family member found a tick on their body in the past year, and 224 (3.0%) reported having found a tick on themselves. When asked whether they were worried about the risk of LD, 55.4% reported concern regarding LD (40.1% had little or no concern and 4.0% did not know).

**Table 1:** Descriptive characteristics of the 10,790 study participants

Variable		n (%)^a^
Sex	Male	4113 (38.1)
	Female	6677 (61.9)
Age group	18-24 yr	342 (3.2)
	25-34 yr	763 (7.1)
	35-44 yr	1524 (14.0)
	45-54 yr	1573 (15.0)
	55-64 yr	2669 (25.0)
	65-74 yr	2587 (24.0)
	75+ yr	1332 (12.0)
Subregions (RLS)	511 - La Pommeraie	809 (7.5)
	512 - Haute-Yamaska	1151 (10.7)
	513 - Memphrémagog	822 (7.6)
	514 - Coaticook	803 (7.4)
	515 - Sherbrooke	3971 (36.8)
	516 - Val Saint-François	813 (7.5)
	517 - Asbestos	803 (7.4)
	518 - Haut-Saint-François	810 (7.5)
	519 - Granit	808 (7.5)
Education	No certificate, diploma or degree	1280 (11.9)
	High-school diploma or equivalent	2477 (23.0)
	Trade school	1196 (11.1)
	College^b^	2338 (21.7)
	University degree	3214 (29.8)
	Other	262 (2.4)
	No response	23 (0.2)

^a^sampling weights are not applied to the proportions

^b^following high school and before university

### Awareness and concerns regarding LD

The proportion of respondents who heard about LD before the survey varied by subregions (e.g. Asbestos 93.3%, La Pommeraie 98.3%, *p* < 0.0001), sex (e.g. male 94.7%, female 97.4%, *p* < 0.0001), age (e.g. 18-24 years 89.1%, 55-64 years 98.0%, *p* < 0.0001); and education (e.g. no diploma 90.4%, High school diploma 98.1%, *p* < 0.0001).

Awareness of a risk of acquiring LD in the respondent’s municipality did not vary by sex but varied significantly by subregions (e.g. Asbestos 58.6%, La Pommeraie 87.9%, *p* < 0.0001), by age (e.g. 18-24 years 72.2%, 35-44 years 85.2% *p* < 0.0001) and education (e.g. no diploma 52.2%, university degree 84.8%, *p* < 0.0001).

The level of concern about acquiring LD varied by subregions (e.g. Asbestos 11.7% very concerned, La Pommeraie 24.1%, *p* < 0.0001), sex (e.g. male 17.1%, female 19.4%, *p* = 0.005), age (e.g. 18-24 years 9.3%, 35-44 years 23.5%, *p* < 0.0001); and education (e.g. university diploma 16.3%, no diploma 21.1%, *p* < 0.0001). Supplementary file 1 presents detailed data for these variables.

### Tick exposure

**Fig. 2:**
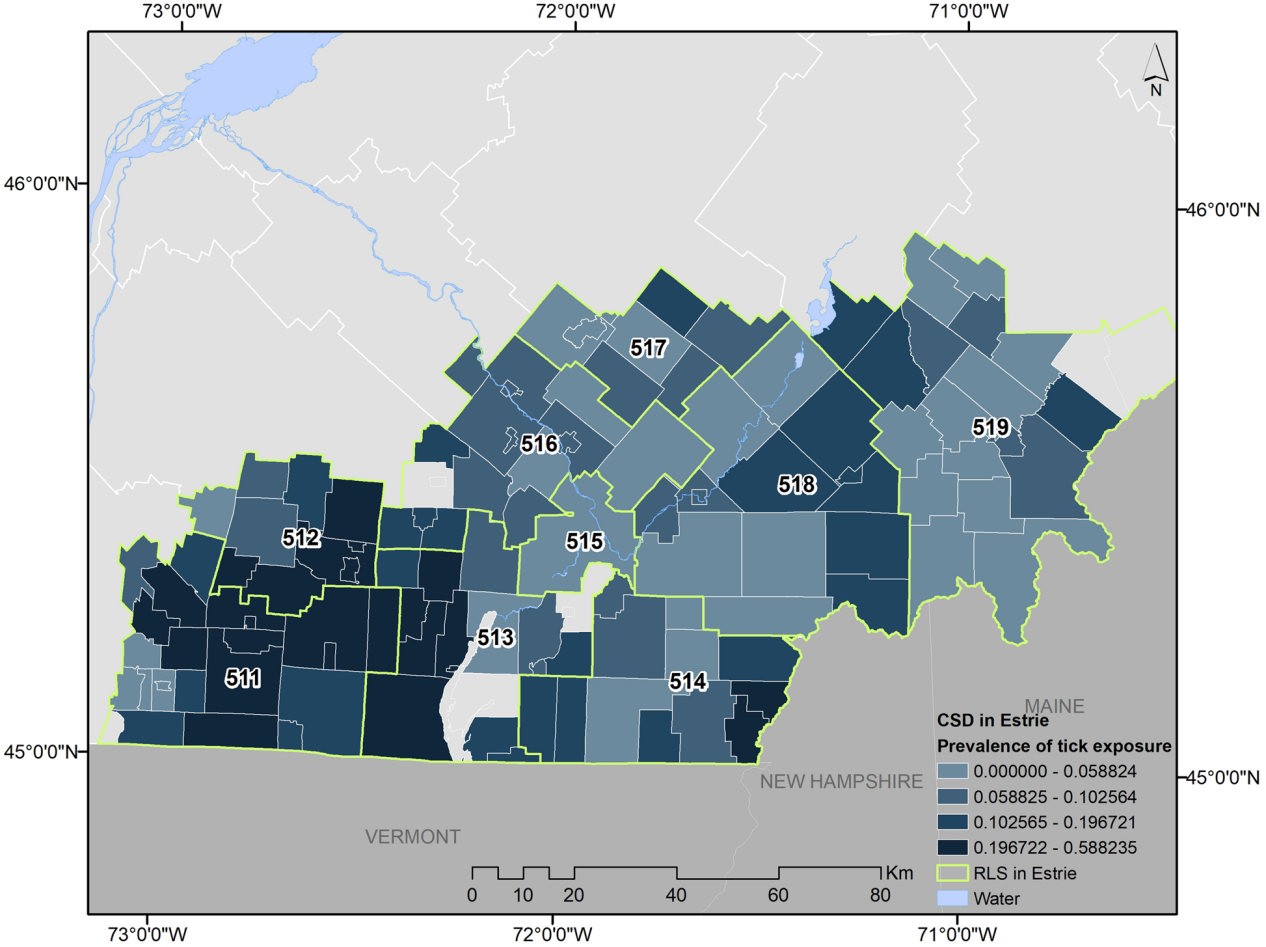
Prevalence of reported tick exposure at the municipality level

The prevalence of reported tick exposure of any household member (including the respondent), during the last 12 months varied by subregions (e.g. Asbestos 3.4%, La Pommeraie 21.9%, *p* < 0.0001), age (e.g. 75+ years 4.5%, 35-44 years 15.1%, *p* < 0.0001); and education (e.g. no diploma 4.9%, Trade school 11.8%, *p* < 0.0001) (Table 2, Fig. 2). When considering the prevalence of respondents finding a tick on themselves during the past 12 months, there was little difference between males and females, though there was variation between age groups (*p* = 0.02) and education levels (*p* < 0.0001). The 35 to 44-year-old group had nearly twice the prevalence relative to the next highest prevalence age group (35 to 44 years = 5.3%, vs 3.0% for 65 to 74 years) and those without a diploma had a lower prevalence than those having high school, trade school, college or university diploma (Table 2).

**Table 2:** Prevalence of reported tick exposure during the past 12 months at the household level (including the respondent), and at the respondent level (respondent only) by subregions, sex, age and education

		Household level		Respondent level	
		n	% (95% CI)*	n	% (95% CI)*
**Subregions (RLS)**	511 - La Pommeraie	179	21.9 (18.4, 25.3)	64	8.2 (5.9, 10.4)
	512 - Haute-Yamaska	151	14.0 (9.70, 18.4)	42	4.3 (1.5, 7.2)
	513 - Memphrémagog	70	8.6 (4.6, 12.6)	22	2.6 (0.1, 5.2)
	514 - Coaticook	61	8.5 (4.4, 12.7)	13	1.9 0.0, 4.4)
	515 - Sherbrooke	191	5.3 (1.7, 8.8)	41	1.2 (0.0, 3.5)
	516 - Val Saint-François	47	6.3 (2.3, 10.2)	13	1.9 (0.0, 4.4)
	517 - Asbestos	30	3.4 (0.0, 7.0)	6	0.7 (0.0, 3.0)
	518 - Haut-Saint-François	42	6.3 (2.2, 10.4)	15	2.6 (0.0, 5.4)
	519 - Granit	38	5.4 (1.5, 9.3)	8	1.5 (0.0, 4.1)
**Sex**	Male	285	9.2 (7.9, 10.4)	113	3.6 (2.8, 4.5)
	Female	524	10.1 (8.4, 11.8)	111	2.4 (1.3, 3.4)
**Age group**	18 - 24 yr	18	7.0 (3.1, 10.9)	4	2.8 (0.0, 6.0)
	25 - 34 yr	85	12.4 (7.4, 17.4)	20	2.7 (0.0, 6.1)
	35 - 44 yr	185	15.1 (10.5, 19.7)	50	5.3 (1.6, 8.9)
	45 - 54 yr	115	9.5 (5.2, 13.8)	34	2.8 (0.0, 6.2)
	55 - 64 yr	191	8.8 (4.7, 12.9)	39	1.9 (0.0, 5.1)
	65 - 74 yr	166	7.6 (3.5, 11.7)	57	3.0 (0.0, 6.4)
	75+yr	49	4.5 (0.3, 8.8)	20	2.2 (0.0, 2.9)
**Education**	No diploma	48	4.9 (3.2, 6.6)	18	1.8 (0.8, 2.8)
	High school diploma	162	8.6 (6.9, 10.3)	46	3.4 (2.1, 4.6)
	Trade school	108	11.8 (9.0, 14.6)	30	3.1 (1.8, 4.5)
	College diploma	213	11.3 (9.5, 13.0)	47	2.7 (1.8, 3.6)
	University degree	269	10.4 (8.8, 11.9)	81	3.4 (2.4, 4.5)
	Other	9	3.0 (0.1, 5.3)	2	0.6 (0.0, 1.3)

*Confidence intervals based on logistic regression with sampling weights

### Preventive behaviors

Preventive behaviors that were assessed included the use of tick repellent, showering and tick checks after visiting LD high-risk areas. Of those for whom the question applied, 30.1% (*n* = 2807) used tick repellent before, 44.6% (*n* = 4116) took a shower after, and 27.0% (*n* = 2484) inspected their skin after visiting a high-LD risk area. Only 10.4% (*n* = 877) adopted all three behaviors regularly. Adoption of preventive behaviors varied by sex and was different for each preventive behavior (e.g. tick repellent in males = 27.3% vs 38.2% in females; shower in male = 51.3% vs 48.7% in female; tick checks in male = 27.1% vs 32.8% in females, all *p* < 0.0001). The 65+ year old respondents had a lower adoption for all preventive behaviors relative to younger age groups. Those residing in the Pommeraie region, the RLS with the highest tick exposure prevalence, had the highest proportion of adoption of preventive behaviors (tick repellent 39.1%, shower 54.2%, tick checks 44.4%). Time spent outdoors in forests, woods or tall grass for primary occupation was significantly related to the likelihood of reporting preventive behaviors (e.g. tick repellent for respondents with 5h hours per day = 39.4%, vs 33.2% for respondents with time spent outside < 1 h per day) (Table 3, Supplementary file 3).

**Table 3:** Number and proportion of respondents reporting tick bites and tick-borne disease preventive behaviors per subregions, sex, age, education and time spent outdoors (except for sex and showering, all differences between categories are statistically significant, with *p* values l < 0.03)

		Repellent		Shower		Tick checks	
		n	%	n	%	n	%
**Subregions**	511 - La Pommeraie	276	39.1	362	54.2	330	44.4
	512 - Haute-Yamaska	339	35.2	471	51.3	347	35.6
	513 - Memphrémagog	244	34.0	353	52.0	231	31.6
	514 - Coaticook	193	27.4	329	49.7	222	31.4
	515 - Sherbrooke	952	29.5	1421	48.1	773	24.6
	516 - Val Saint-François	249	35.5	310	49.1	160	22.8
	517 - Asbestos	151	26.0	226	39.5	96	15.6
	518 - Haut-Saint-François	201	29.1	331	51.6	181	25.9
	519 - Granit	202	32.6	313	49.7	144	22
**Sex**	Male	2835	72.7	1668	51.3	818	27.1
	Female	3772	61.8	2448	48.7	1666	32.8
**Age group**	18 - 24 yr	122	39.4	200	69.3	111	37.5
	25 - 34 yr	319	46	436	64	270	37.2
	35 - 44 yr	683	46	795	56.3	527	37.5
	45 - 54 yr	484	33.1	681	49.8	367	26.8
	55 - 64 yr	653	26.7	1036	46.1	610	27.6
	65 - 74 yr	441	20.6	774	38	474	24.3
	75+ yr	105	12.5	194	23.2	125	14
**Education**	No diploma	149	18.8	344	43.3	162	19.2
	High school diploma	543	29.4	865	48.7	468	28.1
	Trade school	330	35.3	476	50.5	260	29.2
	College diploma	737	37.2	961	51	606	30.8
	University degree	1013	35.4	1386	52	937	33.8
	Other	33	17.3	77	46	46	27
**Time spent outdoors for primary occupation**	5h hours per day	162	39.4	294	63.8	180	42.4
	1-4 h per day	496	37.7	764	57.7	510	39.8
	< 1 h per day	962	33.2	1490	53.8	948	33.2
	no time	1045	29.9	1384	42.7	756	22.7

Among respondents and household members that sought healthcare following a tick bite (108 out of 809, 13.1%), 23.1% (*n* = 25) were given PEP, 20.4% (*n* = 22) received a multiple-day antibiotic prescription, 51.9% (*n* = 56) did not receive a prescription, 2.8% (*n* = 3) do not know, and 1.9% (*n* = 2) did not respond (Supplementary file 4).

### Multivariable analyses

After accounting for the public health risk level, age, education and sex, the only behavior associated with reported tick exposure was performing a tick check after visiting a high-risk area (Table 4). Individuals performing tick checks had higher odds of reporting tick exposure at the individual level. Other significant factors that increased the odds of reported tick exposure were time spent daily outdoors, higher public health risk level, and living in a home located within 500 m of high-risk area (Table 4).

**Table 4:** Associations between reported tick exposure and socio-demographic, environmental and behavioral factors. The table presents results from the multivariable analysis conducted utilising (1) mixed model using random effects for RLS and no weights, and (2) quasi binomial regression with weights (OR = odds ratio; CI = confidence interval). The reference category for the preventive behaviors is either “never” or “rarely”. The reference for the gardening, hiking and camping is no regular engagement in these activities

		Random effects for RLS no weights		Quasi binomial with weights	
		OR	95% CI	OR	95% CI
Intercept		0.0015	0.0005-0.0052	0.0017	0.0004-0.0068
Sex	Male	ref	–	ref	–
	Female	0.58	0.43-0.77	0.68	0.44-1.06
Age	18-24 yr	ref	–	ref	–
	25-34yr	2.08	0.68-6.33	0.92	0.23-3.72
	25-44 yr	2.87	1.00-8.26	1.94	0.52-7.25
	45-54 yr	2.12	0.72-6.2	1.23	0.31-4.90
	55-64 yr	1.39	0.48-4.05	0.81	0.20-3.32
	65-74 yr	2.33	0.81-6.71	1.34	0.34-5.32
	75+ yr	2.60	0.83-8.16	1.85	0.39-8.84
Education	No diploma	ref	–	ref	–
	High school diploma or less	1.07	0.77-1.49	1.23	0.76-2.01
Preventive behaviors	Tick repellent	1.08	0.79-1.46	1.06	0.70-1.59
	Tick checks	4.35	3.14-6.03	4.33	2.59-7.22
	Shower	1.12	0.82-1.52	1.23	0.81-1.89
Daily time spent outdoors	0 h	ref	–	ref	–
	< 1 h	1.06	0.72-1.56	1.08	0.61-1.92
	1-4 hrs	1.97	1.32-2.94	1.83	1.08-3.10
	5h hrs	3.63	2.26-5.83	3.09	1.61-5.92
Outdoor activities	Gardening	1.10	0.80-1.51	1.12	0.76-1.63
	Camping	0.98	0.71-1.36	1.07	0.70-1.64
	Hiking	1.36	0.98-1.88	1.10	0.70-1.72
Home within 500 ft. of high-risk area		1.70	1.10-2.62	2.40	1.41-4.09
Public health risk level		1.85	1.33-2.58	2.14	1.48-3.10

## Discussion

This study investigated the prevalence of reported tick exposure as well as risk and preventive behaviors in a large sample of respondents living in a highly LD endemic region in Canada. Results showed that performing tick checks regularly, spending more time outdoors in forests, woods or tall grass, and living in a region where the LD public health risk level was higher were associated with an increased chance of reporting a tick exposure. No other risk or preventive behaviors were found to be significantly associated with this outcome, although several of them were found to be associated with tick bites or tick-borne disease risk in previous studies [20].

Most importantly, our results show a high level of awareness regarding LD in the study region, but still a low level of adoption of preventive behaviors. This observation is also true for individuals living in subregions considered at significant risk for LD by public health authorities [24]. With only 27% of all respondents performing tick checks after visiting a high-risk area, this study raises concerns regarding the effectiveness of key public health messaging in this highly endemic region. LD risk communication in this region mostly consists of making information on risk and preventive behaviors available on public health authority websites, with occasional articles published in local or general media.

Over the last decade, our team has studied LD prevention in Canada from different perspectives. We examined LD awareness and preventive behaviors at regional [21, 27] and national scales [11, 28]. We previously found that despite the deployment of large scale communication campaigns, the level of adoption of preventive behaviors by the Canadian population remained low [28]. In 2014, less than half of surveyed Canadians who were aware of LD had adopted preventive behaviors toward tick bites, such as regular tick checks (reported by 52% in Canada and 29% when considering residents of the Quebec province only), showering or bathing after possible exposure (41% in Canada, 44% in Quebec), or use of tick repellent (41% in Canada, 47% in Quebec) [11]. This new study unfortunately reveals that the situation has not improved in 4 years, at least in the Estrie region where LD risk is the highest in Quebec.

Limited adoption of preventive behaviors have also been documented in other countries with endemic LD [29–33]. Factors associated with the adoption of these preventive behaviors have been studied in several contexts and vary from one study to another, but some are identified more frequently: good knowledge about LD, high risk perception, a strong perception that it is possible to protect oneself against the disease, and high perceived efficacy of the behavior in question [21, 29, 30, 34, 35]. All these factors could, and should, be targeted by public health communication programs in tick-borne disease endemic regions.

So, what could be done to increase the adoption of preventive behaviors in high-risk populations? We suggest three areas where improvements could be made in order to better promote the adoption of preventive behaviors in exposed populations and to document their effectiveness.

First, there is a need to revisit educational and communication programs targeting tick bite and tick-borne disease prevention in Canada. Conventional top-down risk-reduction strategies such as large-scale communication campaigns are not sufficient to achieve the necessary changes in LD preventive behaviors within high-risk populations. Only a few studies have successfully documented the effect of educational interventions in The Netherlands and in the United States [32, 33]. Innovative approaches have been developed and implemented in other countries, including school-based interventions, the use of video games and mobile phone applications [36–38]. These approaches have in common a foundational, strong theoretical model for behavior change. These innovations have shown promising results to increase knowledge, attitudes and preventive behaviors and could serve as models for improving communication interventions to prevent tick-bites and LD in Canada.

Second, more research is needed to strengthen evidence on the effectiveness and cost-effectiveness of communication interventions to prevent tick bites and tick-borne diseases. The current quality of available evidence has been found to be low in a recent systematic review, making it difficult to convince decision-makers to invest resources in the development of novel communication tools [39]. A better understanding of barriers to adoption of preventive behaviors in Canada is also needed and would provide important insights to better adapt future communications.

Finally, we believe that public health authorities need to monitor the evolution of tick-borne disease preventive behaviors in the Canadian population. Surveillance resources are currently mostly invested in acarological surveillance, which should be maintained over time to monitor the trends of tick populations and tick-borne pathogens. However, we argue that surveillance programs could additionally collect longitudinal data on risk and preventive behaviors, as it is the only way to assess the adaptation of the Canadian population to this emerging threat over time.

This study has limitations. The cross-sectional design of the study cannot consider the temporality of events (respondents may have been exposed to ticks before adopting risk and preventive behaviors) and results should be interpreted cautiously. Another limitation is the use of a survey to assess self-reported tick exposure and behaviors. Self-reported risk and preventive behaviors data can be affected by recall and desirability bias. Reported tick exposure certainly represents an underestimation of the true exposure to ticks and tick-borne diseases in the study region. Human tick encounters have been found to be a robust indicator of tick-borne disease risk in the United States, but self-reported tick exposure as an indicator of tick-borne disease risk has yet to be studied within the Canadian context, where inhabitants are less aware of ticks [40]. Finally, the total number of questions included for the purpose of this project was restricted because they were part of a larger health population survey. Consequently, we could not investigate some important factors that are known to be drivers of preventive behaviors, such as risk perception and the perceived effectiveness of these behaviors [21, 41]. More research is needed to investigate how these factors are changing in the Canadian context in order to better inform communication strategies.

## Conclusion

This study is the first to report on risk and preventive behaviors associated with tick exposure in a population living in a highly LD endemic region in Canada. This study highlights the low level of adoption of preventive behaviors against tick bites in a region where LD risk is amongst the highest in Canada. This suggests a lack of improvement in LD prevention, as low levels of adoption were already reported in studies conducted in the last decade. Innovative and evidence-based approaches to education and communication on ticks and tick-borne diseases are urgently needed in Canada to address this concerning issue.

## Supplementary Information

**Additional file 1.** Questionnaire.

**Additional file 2: Table S2.1**. Knowledge of Lyme Disease. **Table S2.2**. Awareness of LD risk in municipality of residence. **Table S2.3**. Level of concern.

**Additional file 3: Table S3.1**. Preventive behaviors.

**Additional file 4: Table S4.1**. Antibiotic prescriptions.

## Data Availability

The data that support the findings of this study are available from CIUSSS de l’Estrie-CHUS but restrictions apply to the availability of these data, which were used under license for the current study, and so are not publicly available. Data are however available from the corresponding author upon reasonable request and with permission of CIUSSS de l’Estrie-CHUS.
