# Supplementary material for: Behavioral risk factors associated with reported tick exposure in a Lyme disease high incidence region in Canada
Source: BMC Public Health. 2022 Apr 22;22:807. doi: 10.1186/s12889-022-13222-9 (PMC9027878; doi:10.1186/s12889-022-13222-9)
Supplement: Supplementary file 1 — Additional file 1. Questionnaire. [file 12889_2022_13222_MOESM1_ESM.pdf]

Questionnaire

1. **Have you ever heard of Lyme disease?**

   Yes 1
   No 2

2. **Do you believe it is possible to contract Lyme disease in your municipality?**

   Yes 1
   No 2
   I don't know 8
   I prefer not to answer 9

3. **In the last twelve months, have you ever found a tick on yourself or on someone from your family?**

   Yes 1
   No 2
   I prefer not to answer 9
   If yes at 3.1

   3.1 Who was bitten?

   I was bitten by one/many ticks 1
   A member of my family was bitten by one/many ticks 2
   A member of my family and I were bitten by one/many ticks 3
   I prefer not to answer 9
   If 1 or 3 at 3.2

   3.2 Have you consulted a health care professional about this/these tick bite(s)?
   Yes 1
   No 2
   I prefer not to answer 9
   If yes at 3.3

   3.3 Did you receive antibiotics following this/these tick bite(s)?

   Yes, I received one dose of antibiotics to take in one day 1
   Yes, I received antibiotics to take on several days 2
   No 3
   I don't know 4
   I prefer not to answer 9
   If 2 or 3 at 3.4

   3.4 Has the person who was bitten consulted a health professional about this/these tick bite(s)?

   Yes 1
   No 2
   I prefer not to answer 9
   If yes at 3.5

3.5 Has the person who was bitten received antibiotics as a result of this/these bite(s)?

Yes, he/she received one dose of antibiotics to take in one day 1
Yes, he/she received antibiotics to take on several days 2
No 3
I don't know 4
I prefer not to answer 9

4. What is your level of agreement with the following statement: I am worried about contracting Lyme Disease

Strongly agree 1
Somewhat agree 2
Somewhat disagree 3
Strongly disagree 4
I don't know 8
I prefer not to answer 9

5. As part of your primary occupation (work, studies, other), how much time do you spend outdoors in forests, woods or tall grass?

5 hours or more per day 1
One hour to less than five hours per day 2
Less than one hour per day 3
Never 4
Does not apply 8
I prefer not to answer 9

6. Do you regularly do the following activities?

6.1 Gardening

Yes 1
No 2

6.2 Hiking

Yes 1
No 2

6.3 Camping

Yes 1
No 2

6.4 Other outdoor activities

Yes 1
No 2

7. **Do you live near (within 500 feet or 150 meters) an area where there are forests, woods or tall grass?**

Yes 1
No 2

8. Questions 8.1 to 8.3 : In the last twelve months, have you :

8.1 Used insect repellents (mosquito repellent) when you went to a place where there are forests, woods or tall grass to protect yourself from Lyme disease?

Never 1
Rarely 2
Often 3
Always 4
Does not apply 9

8.2 Taken a shower or bath after going to a place where there are forests, woods or tall grass in order to protect yourself from Lyme disease?

Never 1
Rarely 2
Often 3
Always 4
Does not apply 9

8.3 Looked for ticks on your body after going to a place where there are forests, woods or tall grass, in order to protect yourself from Lyme disease?

Never 1
Rarely 2
Often 3
Always 4
Does not apply 9
