# Supplementary material for: Behavioral risk factors associated with reported tick exposure in a Lyme disease high incidence region in Canada
Source: BMC Public Health. 2022 Apr 22;22:807. doi: 10.1186/s12889-022-13222-9 (PMC9027878; doi:10.1186/s12889-022-13222-9)
Supplement: Supplementary file 2 — Additional file 2: Table S2.1. Knowledge of Lyme Disease. Table S2.2. Awareness of LD risk in municipality of residence. Table S2.3. Level of concern. [file 12889_2022_13222_MOESM2_ESM.pdf]

## Supplementary material 2

## Table S2.1 Knowledge of Lyme Disease

| Age | Yes (n) | Yes (%) | No (n) | No (%) | *P* value |
|---|---|---|---|---|---|
| 18 - 24 | 303 | 89.10 | 39.00 | 10.90 | |
| 25 - 34 | 728 | 94.80 | 35.00 | 5.20 | |
| 35 - 44 | 1489 | 97.80 | 35.00 | 2.20 | |
| 45 - 54 | 1520 | 96.80 | 53.00 | 3.20 | |
| 55 - 64 | 2608 | 98.00 | 61.00 | 2.00 | |
| 65 - 74 | 2530 | 97.70 | 67.00 | 2.30 | |
| 75+ | 1242 | 93.50 | 90.00 | 6.50 | <0.0001 |
| **Sex** | | | | | |
| Male | 3900 | 94.70 | | 5.30 | |
| Female | 6510 | 97.40 | | 2.60 | <0.0001 |
| **RLS** | | | | | |
| RLS 511 la Pommeraie | 794 | 98.30 | 15 | 1.70 | |
| RLS 512 la Haute-Yamaska | 1132 | 98.20 | 19 | 1.80 | |
| RLS 513 Memphrémagog | 809 | 98.20 | 13 | 1.80 | |
| RLS 514 Coaticook | 788 | 98.20 | 15 | 1.80 | |
| RLS 515 Sherbrooke | 3790 | 93.50 | 181 | 6.50 | |
| RLS 516 Val Saint-François | 791 | 96.50 | 22 | 3.50 | |
| RLS 517 Asbestos | 753 | 93.20 | 50 | 6.80 | |
| RLS 518 Haut-Saint-François | 788 | 96.40 | 22 | 3.60 | |
| RLS 519 Granit | 765 | 93.30 | 43 | 6.70 | <0.0001 |
| **Education** | | | | | |
| No response | 21 | 84.10 | 2 | 15.90 | |
| No diploma | 1166 | 90.40 | 114 | 9.60 | |
| Other | 2386 | 95.70 | 91 | 4.30 | |
| High School diploma | 1172 | 98.10 | 24 | 1.90 | |
| College diploma | 2282 | 96.70 | 56 | 3.30 | |
| University degree | 3136 | 97.10 | 78 | 2.90 | |
| Trade school | 247 | 95.10 | 15 | 4.90 | <0.0001 |

## Table S2.2 Awareness of risk of LD acquisition in municipality of residence

| Age | Aware (n) | Aware (%) | Not aware (n) | Not aware (%) | Do not know (n) | Do not know (%) | *P* value |
|---|---|---|---|---|---|---|---|
| 18 - 24 | 217 | 72.20 | 45 | 15.3 | 41 | 12.5 | |
| 25 - 34 | 594 | 82.20 | 59 | 7 | 75 | 10.8 | |
| 35 - 44 | 1248 | 85.20 | 108 | 6.8 | 132 | 8 | |
| 45 - 54 | 1201 | 81.20 | 165 | 9.8 | 154 | 9 | |
| 55 - 64 | 1867 | 74.50 | 406 | 14.1 | 333 | 11.4 | |
| 65 - 74 | 1681 | 72.30 | 465 | 15.3 | 370 | 12.4 | |
| 75+ | 619 | 52.20 | 356 | 27.7 | 263 | 20.1 | <0.0001 |
| **Sex** | | | | | | | |
| Male | 2785 | 75.20 | 604 | 12.90 | 509 | 11.80 | |
| Female | 4642 | 75.30 | 1000 | 13.30 | 859 | 11.40 | 0.8177 |
| **RLS** | | | | | | | |
| RLS 511 la Pommeraie | 710 | 87.90 | 38 | 5.20 | 46 | 6.90 | |
| RLS 512 la Haute-Yamaska | 926 | 82.40 | 106 | 9.50 | 100 | 8.10 | |
| RLS 513 Memphrémagog | 625 | 77.00 | 100 | 11.90 | 83 | 11.10 | |
| RLS 514 Coaticook | 596 | 76.40 | 91 | 11.00 | 100 | 12.60 | |
| RLS 515 Sherbrooke | 2693 | 72.20 | 605 | 15.30 | 488 | 12.60 | |
| RLS 516 Val Saint-François | 516 | 67.60 | 146 | 16.50 | 127 | 15.90 | |
| RLS 517 Asbestos | 414 | 58.60 | 184 | 21.80 | 155 | 19.60 | |
| RLS 518 Haut-Saint-François | 504 | 64.70 | 144 | 18.40 | 138 | 16.90 | |
| RLS 519 Granit | 443 | 58.80 | 190 | 23.80 | 131 | 17.50 | <0.0001 |
| **Education** | | | | | | | |
| No response | 8 | 35.80 | 5 | 20.90 | 8 | 43.30 | |
| No diploma | 616 | 59.20 | 306 | 23.10 | 238 | 17.70 | |
| High school diploma | 1527 | 68.00 | 453 | 16.70 | 403 | 15.40 | |
| Trade school | 837 | 75.00 | 168 | 11.00 | 166 | 14.00 | |
| College diploma | 1744 | 78.90 | 297 | 11.70 | 241 | 9.40 | |
| University degree | 2567 | 84.80 | 311 | 8.50 | 257 | 6.70 | |
| Other | 128 | 56.00 | 64 | 22.20 | 55 | 21.80 | <0.0001 |

## Table S2.3 Level of concern

| Age | Very concer-ned (n) | Very concern-ed (%) | Concerned(n) | Concerned(%) | Somewhat Concerned(n) | Somewhat Concerned (%) | Not concerned (n) | Not concerned (%) | Don't know (n) | Don't know (%) | ***P* value** |
|---|---|---|---|---|---|---|---|---|---|---|---|
| 18 - 24 | 32 | 9.3 | 120 | 40.1 | 103 | 34.8 | 38 | 13.1 | 9 | 2.7 | |
| 25 - 34 | 165 | 21.7 | 300 | 42.2 | 163 | 21.5 | 77 | 10.6 | 23 | 3.9 | |
| 35 - 44 | 321 | 23.5 | 649 | 43 | 346 | 21.3 | 130 | 9.7 | 39 | 2.5 | |
| 45 - 54 | 287 | 19.9 | 590 | 38.3 | 376 | 24.3 | 197 | 13.6 | 64 | 4 | |
| 55 - 64 | 498 | 20.4 | 972 | 36.7 | 638 | 24.2 | 388 | 14.9 | 102 | 3.7 | |
| 65 - 74 | 379 | 15.6 | 879 | 35.7 | 632 | 25.1 | 482 | 19.2 | 125 | 4.4 | |
| 75+ | 131 | 10.3 | 275 | 23.4 | 308 | 25.4 | 400 | 33.4 | 103 | 7.5 | <0.0001 |
| **Sex** | | | | | | | | | | | |
| Male | 603 | 17.1 | 1335 | 36.5 | 1026 | 25.1 | 739 | 17.2 | 176 | 4.1 | |
| Female | 1210 | 19.4 | 2450 | 38.3 | 1540 | 23.9 | 973 | 14.4 | 289 | 3.9 | 0.0049 |
| **RLS** | | | | | | | | | | | |
| 511 | 209 | 24.1 | 281 | 36.7 | 163 | 19.8 | 111 | 15.4 | 25 | 3.9 | |
| 512 | 247 | 22.5 | 436 | 39.1 | 227 | 20.8 | 171 | 14.5 | 45 | 3.1 | |
| 513 | 152 | 17.8 | 323 | 41.1 | 185 | 22.3 | 116 | 15.1 | 29 | 3.7 | |
| 514 | 161 | 21.4 | 306 | 38.4 | 179 | 23.9 | 91 | 11.6 | 42 | 4.7 | |
| 515 | 553 | 14.4 | 1337 | 35.7 | 1032 | 28.8 | 674 | 17 | 173 | 4.2 | |
| 516 | 129 | 17.3 | 300 | 37.1 | 200 | 25.9 | 118 | 13.7 | 41 | 6 | |
| 517 | 102 | 11.7 | 281 | 41.2 | 182 | 25.2 | 136 | 16.8 | 43 | 5.1 | |
| 518 | 136 | 18.2 | 264 | 33.8 | 205 | 26.9 | 143 | 17 | 34 | 4.1 | |
| 519 | 124 | 15.7 | 257 | 35.8 | 193 | 24.4 | 152 | 20.1 | 33 | 4.2 | <0.0001 |
| **Education** | | | | | | | | | | | |
| No response | 3 | 18.4 | 7 | 30.8 | 4 | 13.2 | 5 | 29.7 | 1 | 7.9 | |
| No diploma | 231 | 21.1 | 354 | 30.4 | 210 | 18.3 | 263 | 22.9 | 86 | 7.3 | |
| High school diploma | 411 | 18.6 | 870 | 36.8 | 543 | 22.2 | 389 | 16.4 | 157 | 5.9 | |
| Trade school | 233 | 20.5 | 447 | 40 | 252 | 21.4 | 179 | 13.6 | 53 | 4.6 | |
| College diploma | 400 | 17.8 | 861 | 38.9 | 604 | 26.6 | 329 | 14 | 80 | 2.8 | |
| University degree | 483 | 16.3 | 1190 | 39.1 | 901 | 28 | 487 | 14.9 | 64 | 1.8 | |
| Other | 52 | 23.4 | 56 | 22.7 | 52 | 22.9 | 60 | 20.7 | 24 | 10.3 | <0.0001 |
