# Supplementary material for: Behavioral risk factors associated with reported tick exposure in a Lyme disease high incidence region in Canada
Source: BMC Public Health. 2022 Apr 22;22:807. doi: 10.1186/s12889-022-13222-9 (PMC9027878; doi:10.1186/s12889-022-13222-9)
Supplement: Supplementary file 3 — Additional file 3: Table S3.1. Preventive behaviors. [file 12889_2022_13222_MOESM3_ESM.pdf]

Supplementary material 3

Table S3.1 Preventive behaviors

INSECT REPELLENT	Yes (n)	Yes (%)	No (n)	No (%)	***P* value**
**Age**					
18 - 24	122	39.4	173	60.6	
25 - 34	319	46	390	54	
35 - 44	683	46	771	54	
45 - 54	484	33.1	952	66.9	
55 - 64	653	26.7	1747	73.3	
65 - 74	441	20.6	1736	79.4	
75+	105	12.5	838	87.5	<0.0001
**Sex**					
Male	2835	72.7	836	27.3	
Female	3772	61.8	1971	38.2	<0.0001
**RLS**					
511	276	39.1	456	60.9	
512	339	35.2	675	64.8	
513	244	34	502	66	
514	193	27.4	536	72.6	
515	952	29.5	2432	70.5	
516	249	35.5	474	64.5	
517	151	26	506	74	
518	201	29.1	520	70.9	
519	202	32.6	506	67.4	<0.0001
**Education**					
No response	2	5.3	14	94.7	
No diploma	149	18.8	808	81.2	
High school diploma	543	29.4	1561	70.6	
Trade school	330	35.3	738	64.7	
College diploma	737	37.2	1381	62.8	
University degree	1013	35.4	1949	64.6	
Other	33	17.3	156	82.7	<0.0001
**Occupation time high risk areas**					
5+ hours per day	162	39.4	300	60.6	
1-4 hours per day	496	37.7	914	62.3	
<1 hour per day	962	33.2	2142	66.8	
no time	1045	29.9	2903	70.1	0.00018

SHOWER AFTER	Yes (n)	Yes (%)	No (n)	No (%)	***P* value**
**Age**					
18 - 24	200	69.3	94	30.7	
25 - 34	436	64	265	36	
35 - 44	795	56.3	640	43.7	
45 - 54	681	49.8	706	50.2	
55 - 64	1036	46.1	1280	53.9	
65 - 74	774	38	1293	62	
75+	194	23.2	667	76.8	p<0.0001
**Sex**					
Male	1668	51.3	1895	48.7	
Female	2448	48.7	3050	51.3	0.0613
**RLS**					
511	362	54.2	344	45.8	
512	471	51.3	512	48.7	
513	353	52	370	48	
514	329	49.7	393	50.3	
515	1421	48.1	1822	51.9	
516	310	49.1	383	50.9	
517	226	39.5	386	60.5	
518	331	51.6	368	48.4	
519	313	49.7	367	50.3	0.0017
**Education**					
No response	7	62.5	7	37.5	
No diploma	344	43.3	562	56.7	
High school diploma	865	48.7	1153	51.3	
Trade school	476	50.5	549	49.5	
College diploma	961	51	1080	49	
University degree	1386	52	1488	48	
Other	77	46	106	54	0.0282
**Occupation time high risk areas**					
5+ hours per day	294	63.8	167	36.2	
1-4 hours per day	764	57.7	627	42.3	
<1 hour per day	1490	53.8	1558	46.2	
no time	1384	42.7	2341	57.3	
Not applicable	169	45.6	232	54.4	
No response	15	46.2	20	53.8	<0.0001

TICK CHECK	Yes (n)	Yes (%)	No (n)	No (%)	***P* value**
**Age**					
18 - 24	111	37.5	183	62.5	
25 - 34	270	37.2	439	62.8	
35 - 44	527	37.5	921	62.5	
45 - 54	367	26.8	1041	73.2	
55 - 64	610	27.6	1741	72.4	
65 - 74	474	24.3	1630	75.7	
75+	125	14	760	86	<0.0001
**Sex**					
Male	818	27.1	2797	72.9	
Female	1666	32.8	3918	67.2	<0.0001
**RLS**					
511	330	44.4	386	55.6	
512	347	35.6	647	64.4	
513	231	31.6	502	68.4	
514	222	31.4	504	68.6	
515	773	24.6	2519	75.4	
516	160	22.8	553	77.2	
517	96	15.6	530	84.4	
518	181	25.9	525	74.1	
519	144	22	549	78	<0.0001
**Education**					
No response	5	40.6	9	59.4	
No diploma	162	19.2	752	80.8	
High school diploma	468	28.1	1588	71.9	
Trade school	260	29.2	777	70.8	
College diploma	606	30.8	1477	69.2	
University degree	937	33.8	1974	66.2	
Other	46	27	138	73	<0.0001
**Occupation time high risk areas**					
5+ hours per day	180	42.4	283	57.6	
1-4 hours per day	510	39.8	895	60.2	
<1 hour per day	948	33.2	2139	66.8	
no time	756	22.7	3034	77.3	
Not applicable	84	23.5	336	76.5	
No response	6	13.5	28	86.5	<0.0001
