# Supplementary material for: Behavioral risk factors associated with reported tick exposure in a Lyme disease high incidence region in Canada
Source: BMC Public Health. 2022 Apr 22;22:807. doi: 10.1186/s12889-022-13222-9 (PMC9027878; doi:10.1186/s12889-022-13222-9)
Supplement: Supplementary file 4 — Additional file 4: Table S4.1. Antibiotic prescriptions. [file 12889_2022_13222_MOESM4_ESM.pdf]

Supplementary material 4

Table S4.1 Antibiotic prescriptions

Age	One day (n)	One day (%)	Multiple days (n)	Multiple days (%)	None (n)	None (%)	Don't know (n)	Don't know (%)
25 - 34	1	7.5	1	30	6	62.5	0	0
35 - 44	3	18.7	7	28.8	11	52.5	0	0
45 - 54	4	20.5	4	32	9	47.5	0	0
55 - 64	5	34.2	3	6.8	10	59.1	0	0
65 - 74	10	35.2	5	17.9	16	42.2	2	4.8
75+	2	19	2	27.3	4	35.2	1	18.5
**Sex**								
Male	12	22.9	13	26.9		49.4		0.8
Female	13	23.4	9	18.9		53.2		4.5
**RLS**								
511	8	16.1	11	32.9	19	47.3	2	3.6
512	7	34.8	4	22.9	9	42.3	0	0
513	3	36.4	0	0	6	63.6	0	0
514	1	6.5	2	43.5	4	50	0	0
515	5	23	2	6.4	9	65.2	1	5.4
516	1	13.9	0	0	3	86.1	0	0
517	0	0	0	0	1	100	0	0
518	0	0	3	53	3	47	0	0
519	0	0	0	0	2	100	0	0
**Education**								
No diploma	2	20.4	3	40.5	4	39.1	0	0
High school diploma	2	16.5	7	39.1	12	40.1	1	4.3
Trade school	2	17	2	4.9	9	78.1	0	0
College diploma	4	20.6	4	18.3	12	58.3	1	2.8
University degree	15	33	6	18.1	19	49	0	0
Other	0	0	0	0	0	0	1	100
